# Association between leukocytes telomere length and parental consanguineous marriage

**DOI:** 10.17179/excli2024-7920

**Published:** 2025-01-17

**Authors:** Fatemeh Zahra Darvishi, Mostafa Saadat

**Affiliations:** 1 Department of Biology, School of Science, Shiraz University, Shiraz 71467-13565, Iran

## ⁯

Consanguineous marriage (CM, marriage between second cousins and closer relatives) is common in many Asian and African countries. There is a positive association between CM and offspring mortality. There is a negative association between healthy life expectancy at birth (HALE) and the national mean of CM (Saadat, 2011[8]).

Today, telomere length is used as a biological marker for aging. Critically, short telomeres are associated with aging and ultimately death of cells. Accumulative evidence suggests that telomere erosion is associated with aging, mortality and aging-related diseases (Gruber et al., 2021[6]). Both telomere shortening and consanguinity are associated with the risk of many multifactorial diseases. Therefore, it could be suggested that parental CM may be an independent factor associated with telomere attrition and subsequently with age-related diseases and HALE. We know that both inbreeding and telomere length are significantly associated with higher mortality, lower life expectancy at birth, and several diseases. These associations do not necessarily imply that inbreeding reduces telomere length. The relationship between telomere length and parental consanguinity remains understudied. For populations such as ours, with a high level of consanguinity, this association is of great importance. Therefore, the present cross-sectional study was conducted in Shiraz (Fars Province, Iran). 

Telomere length and telomere shortening are associated with gender and ethnicity (Barrett and Richardson, 2011[2]; Brown et al., 2017[3]). Therefore, only male participants belonging to Caucasian Muslims living in Shiraz were included in the study. Age, type of parental marriage, and history of alcohol consumption, smoking, and dependence on illicit drugs were collected by interview. The type of parental marriage was divided into two categories: first cousins and unrelated marriages; other types of marriages were excluded from the study. Numerous studies have shown that alcohol consumption (Maugeri et al., 2021[7]), smoking (Astuti et al., 2017[1]) and dependence on illegal drugs (such as heroin) (Darvishi and Saadat, 2022[4]) are associated with telomere length. As these factors may act as confounders, participants with a history of smoking, illegal drug use and alcohol consumption were excluded from the study. All participants were free of diagnosed cancer, cardiovascular disease, asthma, diabetes, schizophrenia and bipolar disorder.

Using G*Power software (version 3.1.9.2), to detect a significant difference in relative telomere length between our study groups (unrelated vs. first cousin marriages) with a power of 0.80, α=0.05 (two-sided), effect size=0.65 (medium to large effect size), allocation ratio of study groups=2.50; a minimum of 94 samples would be required. The Ethics Committee of our University approved the present study (IR.US.PSYEDU.REC.1403.007) and participants gave their informed consent. A total of 166 male subjects were initially selected for the study, and after applying the inclusion and exclusion criteria, 119 participants remained in the study. Of these, 33 (27.7 %) and 86 (72.3 %) were offspring of first cousins and unrelated marriages, respectively. The mean (±SE) inbreeding coefficient among our participants was 0.0173±0.0026. The mean (±SE) age was 31.8±1.34 and 31.9±0.84 years for first cousin and unrelated offspring, respectively, with a similar distribution (t=0.060, df=117, p=0.952).

Relative telomere length (RTL) was measured using genomic DNA extracted from leukocytes as previously described (Darvishi and Saadat, 2021[5]). The mean (±SE) value of RTL was 5.62±0.43 and 6.17±0.33 in leukocytes from offspring of first cousin and unrelated marriages, respectively, which showed no statistical difference (t=0.924, df=117, p=0.357). Correlation analysis was used to examine the relationship between participant age and telomere length, which showed a borderline association between these two variables (r=-0.174, df=117, p=0.059). To remove the potential confounding effects of age on RTL, multivariable linear regression analysis was used (Supplementary information, Table S1). The regression model showed that subjects' age was marginally associated with RTL (p=0.059), whereas parental consanguinity was not associated with RTL (0.346). This result does not support our research hypothesis. On the other hand, it is inconsistent with the findings of previous studies that reported that CM increases the risk of age-related diseases, as well as the finding of a study that showed an inverse relationship between inbreeding coefficient and life expectancy (Saadat, 2011[8]).

This study was carried out in an Islamic country. It is clear that there is a possibility that participants may not admit to using items whose use is forbidden by the Muslim faith. Although participants with a positive history of alcohol use were excluded from the study, it is still likely that some participants may have concealed their alcohol use. 

See also the supplementary data.

## Conflict of interest

None.

## Supplementary Material

Supplementary information

Supplementary data
