# Supplementary material for: Association between leukocytes telomere length and parental consanguineous marriage
Source: EXCLI J. 2025 Jan 17;24:177–8. doi: 10.17179/excli2024-7920 (PMC11830915; doi:10.17179/excli2024-7920)
Supplement: Supplementary information [file EXCLI-24-177-s-001.pdf]

EXCLI Journal 2025;24:177-178 – ISSN 1611-2156
Received: October 11, 2024, accepted: January 15, 2025, published: January 17, 2025

Supplementary information to:

Letter to the editor:

ASSOCIATION BETWEEN LEUKOCYTES TELOMERE LENGTH AND PARENTAL CONSANGUINEOUS MARRIAGE

Fatemeh Zahra Darvishi, Mostafa Saadat*

Department of Biology, School of Science, Shiraz University, Shiraz 71467-13565, Iran

* **Corresponding author**: Mostafa Saadat, Department of Biology, School of Science, Shiraz University, Shiraz 71467-13565, Iran. Fax: +98-71-32280916;
E-mail: saadat@shirazu.ac.ir

https://dx.doi.org/10.17179/excli2024-7920

This is an Open Access article distributed under the terms of the Creative Commons Attribution License (http://creativecommons.org/licenses/by/4.0/).

Table S1: Result of multivariable linear regression analysis of relative telomere length (dependent variable) vs parental consanguinity and age of participants (independent variables)

	Unstandardized coefficients		Standardized coefficients	t	*p*
	B	SE	β		
**Constant**	8.238	1.127	-	7.31	<0.001
**Parental consanguinity***	- 0.277	0.293	- 0.086	- 0.94	0.346
**Age**	- 0.065	0.034	- 0.174	- 1.91	0.059

*Unrelated and first cousin marriages were coded 0 and 1, respectively.
For fitted model: F=2.26, df=2, 116; p=0.109
