# Supplementary material for: Association between leukocytes telomere length and parental consanguineous marriage
Source: EXCLI J. 2025 Jan 17;24:177–8. doi: 10.17179/excli2024-7920 (PMC11830915; doi:10.17179/excli2024-7920)
Supplement: Supplementary data [file EXCLI-24-177-s-002.pdf]

EXCLI Journal 2025;24:177-178 – ISSN 1611-2156
Received: October 11, 2024, accepted: January 15, 2025, published: January 17, 2025

Supplementary data to:

Letter to the editor:

ASSOCIATION BETWEEN LEUKOCYTES TELOMERE LENGTH AND PARENTAL CONSANGUINEOUS MARRIAGE

Fatemeh Zahra Darvishi, Mostafa Saadat*

Department of Biology, School of Science, Shiraz University, Shiraz 71467-13565, Iran

* **Corresponding author**: Mostafa Saadat, Department of Biology, School of Science, Shiraz University, Shiraz 71467-13565, Iran. Fax: +98-71-32280916;
E-mail: saadat@shirazu.ac.ir

https://dx.doi.org/10.17179/excli2024-7920

This is an Open Access article distributed under the terms of the Creative Commons Attribution License (http://creativecommons.org/licenses/by/4.0/).

EXCLI Journal 2025;24:177-178 – ISSN 1611-2156
Received: October 11, 2024, accepted: January 15, 2025, published: January 17, 2025

Raw Data

ID	Parental Marriages	Gender	Smoking habit	Age	Relative Telomere Length	Alcohol Drink-ing	Drug Abuse/ Dependence
1	Unrelated	Male	Non-smoker	35	4.35	No	No
2	Unrelated	Male	Non-smoker	37	5.35	No	No
4	Unrelated	Male	Non-smoker	32	3.01	No	No
6	Unrelated	Male	Non-smoker	22	8.34	No	No
7	Unrelated	Male	Non-smoker	25	5.43	No	No
9	Unrelated	Male	Non-smoker	28	6.59	No	No
10	FC	Male	Non-smoker	23	3.84	No	No
12	Unrelated	Male	Non-smoker	23	4.53	No	No
13	Unrelated	Male	Non-smoker	39	2.66	No	No
15	Unrelated	Male	Non-smoker	31	4.50	No	No
16	FC	Male	Non-smoker	36	8.63	No	No
18	Unrelated	Male	Non-smoker	28	11.08	No	No
19	Unrelated	Male	Non-smoker	38	3.48	No	No
20	FC	Male	Non-smoker	26	2.17	No	No
22	Unrelated	Male	Non-smoker	21	3.20	No	No
23	FC	Male	Non-smoker	29	4.29	No	No
24	Unrelated	Male	Non-smoker	34	6.32	No	No
25	Unrelated	Male	Non-smoker	41	9.71	No	No
26	Unrelated	Male	Non-smoker	27	2.93	No	No
27	Unrelated	Male	Non-smoker	40	4.08	No	No
28	Unrelated	Male	Non-smoker	22	2.06	No	No
32	FC	Male	Non-smoker	32	3.20	No	No
35	Unrelated	Male	Non-smoker	36	15.35	No	No
39	Unrelated	Male	Non-smoker	34	2.73	No	No
40	Unrelated	Male	Non-smoker	32	8.22	No	No
42	FC	Male	Non-smoker	28	8.51	No	No
43	Unrelated	Male	Non-smoker	35	1.88	No	No
45	Unrelated	Male	Non-smoker	33	2.43	No	No
46	Unrelated	Male	Non-smoker	26	4.11	No	No
47	Unrelated	Male	Non-smoker	32	5.10	No	No
48	Unrelated	Male	Non-smoker	23	4.17	No	No
49	FC	Male	Non-smoker	35	9.65	No	No
50	Unrelated	Male	Non-smoker	27	7.36	No	No
51	FC	Male	Non-smoker	32	3.01	No	No
52	Unrelated	Male	Non-smoker	31	8.22	No	No
57	Unrelated	Male	Non-smoker	29	4.66	No	No
60	Unrelated	Male	Non-smoker	28	10.06	No	No
63	Unrelated	Male	Non-smoker	26	3.73	No	No
64	Unrelated	Male	Non-smoker	26	5.90	No	No
65	Unrelated	Male	Non-smoker	27	3.48	No	No
74	Unrelated	Male	Non-smoker	41	5.90	No	No
76	Unrelated	Male	Non-smoker	23	5.06	No	No
83	Unrelated	Male	Non-smoker	39	9.51	No	No
84	Unrelated	Male	Non-smoker	34	9.06	No	No
85	Unrelated	Male	Non-smoker	38	7.41	No	No
86	Unrelated	Male	Non-smoker	27	8.82	No	No
87	FC	Male	Non-smoker	26	3.71	No	No
89	Unrelated	Male	Non-smoker	40	4.56	No	No
90	FC	Male	Non-smoker	27	5.21	No	No
91	FC	Male	Non-smoker	29	5.90	No	No
92	Unrelated	Male	Non-smoker	41	3.86	No	No
93	Unrelated	Male	Non-smoker	41	4.92	No	No
94	Unrelated	Male	Non-smoker	25	11.88	No	No
95	Unrelated	Male	Non-smoker	22	7.26	No	No
96	Unrelated	Male	Non-smoker	34	7.01	No	No
97	Unrelated	Male	Non-smoker	38	3.51	No	No
98	Unrelated	Male	Non-smoker	30	7.01	No	No
99	Unrelated	Male	Non-smoker	36	7.52	No	No
107	Unrelated	Male	Non-smoker	34	6.41	No	No
115	Unrelated	Male	Non-smoker	32	4.08	No	No
117	Unrelated	Male	Non-smoker	41	5.90	No	No
119	FC	Male	Non-smoker	25	5.62	No	No
124	Unrelated	Male	Non-smoker	36	7.46	No	No
125	Unrelated	Male	Non-smoker	21	6.41	No	No
128	Unrelated	Male	Non-smoker	26	6.50	No	No

EXCLI Journal 2025;24:177-178 – ISSN 1611-2156
Received: October 11, 2024, accepted: January 15, 2025, published: January 17, 2025

ID	Parental Marriages	Gender	Smoking habit	Age	Relative Telomere Length	Alcohol Drink-ing	Drug Abuse/ Dependence
129	Unrelated	Male	Non-smoker	36	6.77	No	No
130	FC	Male	Non-smoker	39	5.24	No	No
135	Unrelated	Male	Non-smoker	35	8.11	No	No
136	Unrelated	Male	Non-smoker	24	10.13	No	No
140	FC	Male	Non-smoker	26	5.54	No	No
2002	Unrelated	Male	Non-smoker	53	5.89	No	No
2007	FC	Male	Non-smoker	25	7.83	No	No
2011	Unrelated	Male	Non-smoker	22	22.47	No	No
2012	Unrelated	Male	Non-smoker	25	5.50	No	No
2015	FC	Male	Non-smoker	25	10.93	No	No
2018	Unrelated	Male	Non-smoker	25	7.84	No	No
2021	Unrelated	Male	Non-smoker	28	10.63	No	No
2030	Unrelated	Male	Non-smoker	31	4.86	No	No
2048	FC	Male	Non-smoker	36	5.86	No	No
2063	Unrelated	Male	Non-smoker	29	7.11	No	No
3005	Unrelated	Male	Non-smoker	28	6.73	No	No
3007	FC	Male	Non-smoker	22	8.94	No	No
3012	Unrelated	Male	Non-smoker	25	6.36	No	No
3014	FC	Male	Non-smoker	32	3.27	No	No
3021	Unrelated	Male	Non-smoker	41	4.89	No	No
3025	Unrelated	Male	Non-smoker	59	5.46	No	No
3028	FC	Male	Non-smoker	26	5.74	No	No
3029	Unrelated	Male	Non-smoker	32	3.86	No	No
3030	Unrelated	Male	Non-smoker	33	5.13	No	No
3033	Unrelated	Male	Non-smoker	29	6.68	No	No
3035	Unrelated	Male	Non-smoker	20	5.78	No	No
3037	FC	Male	Non-smoker	33	12.38	No	No
3040	Unrelated	Male	Non-smoker	35	5.39	No	No
3041	FC	Male	Non-smoker	24	4.99	No	No
3042	FC	Male	Non-smoker	47	6.19	No	No
3043	FC	Male	Non-smoker	55	4.20	No	No
3047	FC	Male	Non-smoker	32	7.21	No	No
3048	Unrelated	Male	Non-smoker	20	8.06	No	No
3049	Unrelated	Male	Non-smoker	40	9.25	No	No
3050	Unrelated	Male	Non-smoker	34	5.24	No	No
3052	Unrelated	Male	Non-smoker	29	5.06	No	No
3054	Unrelated	Male	Non-smoker	31	6.45	No	No
3055	FC	Male	Non-smoker	49	4.08	No	No
3056	Unrelated	Male	Non-smoker	21	9.19	No	No
3059	FC	Male	Non-smoker	32	4.14	No	No
3060	FC	Male	Non-smoker	38	2.07	No	No
3061	FC	Male	Non-smoker	31	3.86	No	No
3065	Unrelated	Male	Non-smoker	21	4.11	No	No
3068	Unrelated	Male	Non-smoker	32	3.53	No	No
3070	Unrelated	Male	Non-smoker	35	4.96	No	No
3071	Unrelated	Male	Non-smoker	30	2.35	No	No
3072	Unrelated	Male	Non-smoker	50	2.58	No	No
3075	Unrelated	Male	Non-smoker	50	4.82	No	No
3076	FC	Male	Non-smoker	34	5.82	No	No
3077	FC	Male	Non-smoker	40	2.57	No	No
3079	Unrelated	Male	Non-smoker	46	4.11	No	No
3081	Unrelated	Male	Non-smoker	26	6.02	No	No
3085	FC	Male	Non-smoker	24	4.89	No	No
3086	FC	Male	Non-smoker	31	5.98	No	No

FC=first cousin marriage
